# Death of Dr. Barnes—Action of Erie County Medical Society

**Published:** 1871-07

**Authors:** 


### Death of Dr. Barnes—Action of Erie County Medical Society.

A special meeting of the Erie County Mendical Society convened at its rooms, June 1st, 1871, at 8 o'clock P. M. The President, Dr. Wm. Gould, occupied the chair, and announced the object of the meeting, to take action on the death of Dr. Josiah Barnes.

Dr. Rochester gave a brief account of the last illness of the deceased, as well as a slight sketch of his general character. He said: Dr Barnes died about one o'clock this morning, after having lingered between life and death the past fortnight; he had been in practice here thirty five or more years, and was such a man that his presence could not but be missed by the profession and the general public. He had practiced his profession with resolution, and at a time, and for several years, when it was almost necessary that he should have abstained from professional duties, and retired from active life. The disease from which he suffered was supposed to be cardiac, but, over a year ago, I became convinced his complaint was hepatic; his right lung was likewise filled with fluid, and these complications were doubtless the cause of his death. Mr. President, on this solemn occasion, and in respect for the memory of our departed friend, I move the appointment of a committee of five to draft resolutions expressive of the feelings of this society on this occasion.

Dr. J. P. White said: It was with no ordinary feeling that he arose to second the motion of Dr. Rochester. That Dr. Barnes and myself had been shoulder to shoulder battling with disease for nearly forty years. That himself, Dr. Winne, and myself, had been longer engaged here in practice than any other members of the Profession. I knew him first in 1832. The summer in which I commenced the practice of my profession. He came here to visit a brother-in-law, Hon. Geo. B. Webster, who was suffering from a painful and protracted illness. He was so well pleased with the place that in the following spring he came here to take up his abode with. and practice his profession among us. Both Dr. Winne and Dr. Barnes commenced the practice of medicine in this city in 1833. Every member of our profession then practising medicine in this city, except myself, has passed away. Our offices were in adjoining building, and for a considerable period after his arrival we were thrown very much together, especially in the second epidemic of cholera in 1834. From that period to this, though we never were remarkably intimate, we never had any professional difference. He was peculiar in attaching himself to no parties or cliques. Dr. Barnes possessed a classical education which was remarkable for the time he entered the profession. He graduated at Yale College among the first in his class. Subsequently studied his profession and received his degree of Doctor in Medicine in Philadelphia. He possessed a remarkably well balanced mind, was a very sound practitioner; he had no hobbies; his patrons were among the first citizens of the town, and his patients became very much attached to him. They as well as the profession of our city will greatly deplore his loss.

The motion of Dr. Rochester prevailed, and the President appointed Drs. J. F. Miner, S. F. Mixer, C. C. Wyckoff, C. C. F. Gay, and P. H. Strong, a committee to draft resolutions.

This committe, after a short absence, returned, and the chairman, Dr. Miner, read the following:

WHEREAS, After a long and painful illness, death has removed from our circle our beloved friend and colleague, Dr. Josiah Barnes, who devoted the energies of his educated, strong and active mind to the faithful cultivation and honorable practice of our profession for nearly half a century, and who, by his worthy example and blameless life and character, had endeared himself, not only to the members of the medical profession, but to a very wide circle of relatives and friends; therefore

*Resolved*, That we learn with the deepest sorrow of the death of our venerable and most highly esteemed friend and associate, Dr. Josiah Barnes, whose life has been cut off at a period of great usefulness, when his large experience and mature judgment were most valuable to the profession and the world, by which providence we are left to mourn the death of an educated, accomplished and faithful physician, an able counselor, a warm hearted and generous friend.

*Resolved*, That we remember his unvarying kindness and courtesy in his relations with his professional associates, and recognize with admiration, his impartial judgment in the questions which, during his earlier years, agitated and estranged his professional associates; and that in view of his whole life and character, we regard his death as a great professional and personal affliction.

*Resolved*, That, in great sorrow, on account of professional, public and personal loss, we extend our deepest sympathies to the family and friends of our deceased member, who are called to mourn the loss of an affectionate husband, father and friend.

*Resolved*, That, in token of our loss and sympathy, we attend his funeral in a body and wear the usual badge of mourning.

*Resolved*, That a copy of these resolutions be transmitted to the family of the deceased, and that they be published in the Buffalo *Medical and Surgical Journal*, and in the daily papers of this city.

J. F. MINER,
S. F. MIXER,
C. C. WYCKOFF,
C. C. F. GAY,
P. H. STRONG.

Dr. Miner accompanied the report with some appropriate remarks, and was followed by Drs. Mixer, Harvey, Ring, Hill, Loomis and others.

On motion, the resolutions were adopted unanimously, after which the meeting adjourned.

M. G. POTTER, M. D., *Secretary*,

